# The *ace-1* Locus Is Amplified in All Resistant *Anopheles gambiae* Mosquitoes: Fitness Consequences of Homogeneous and Heterogeneous Duplications

**DOI:** 10.1371/journal.pbio.2000618

**Published:** 2016-12-05

**Authors:** Benoît S. Assogba, Pascal Milesi, Luc S. Djogbénou, Arnaud Berthomieu, Patrick Makoundou, Lamine S. Baba-Moussa, Anna-Sophie Fiston-Lavier, Khalid Belkhir, Pierrick Labbé, Mylène Weill

**Affiliations:** 1 Institut des Sciences de l'Evolution de Montpellier (UMR 5554, CNRS-UM-IRD-EPHE), Université de Montpellier, Place Eugène Bataillon, Montpellier, France; 2 Institut Régional de Santé Publique, Université d’Abomey Calavi, Cotonou, Benin; 3 Faculté des Sciences et Techniques, Laboratoire de Biologie et de Typage Moléculaire en Microbiologie, Université d’Abomey Calavi, Cotonou, Bénin; The Institute of Science and Technology Austria, Austria

## Abstract

Gene copy-number variations are widespread in natural populations, but investigating their phenotypic consequences requires contemporary duplications under selection. Such duplications have been found at the *ace-1* locus (encoding the organophosphate and carbamate insecticides’ target) in the mosquito *Anopheles gambiae* (the major malaria vector); recent studies have revealed their intriguing complexity, consistent with the involvement of various numbers and types (susceptible or resistant to insecticide) of copies. We used an integrative approach, from genome to phenotype level, to investigate the influence of duplication architecture and gene-dosage on mosquito fitness. We found that both heterogeneous (i.e., one susceptible and one resistant *ace-1* copy) and homogeneous (i.e., identical resistant copies) duplications segregated in field populations. The number of copies in homogeneous duplications was variable and positively correlated with acetylcholinesterase activity and resistance level. Determining the genomic structure of the duplicated region revealed that, in both types of duplication, *ace-1* and 11 other genes formed tandem 203kb amplicons. We developed a diagnostic test for duplications, which showed that *ace-1* was amplified in all 173 resistant mosquitoes analyzed (field-collected in several African countries), in heterogeneous or homogeneous duplications. Each type was associated with different fitness trade-offs: heterogeneous duplications conferred an intermediate phenotype (lower resistance and fitness costs), whereas homogeneous duplications tended to increase both resistance and fitness cost, in a complex manner. The type of duplication selected seemed thus to depend on the intensity and distribution of selection pressures. This versatility of trade-offs available through gene duplication highlights the importance of large mutation events in adaptation to environmental variation. This impressive adaptability could have a major impact on vector control in Africa.

## Author Summary

Mutations, whether they affect single nucleotides or large genomic regions, are responsible for the genetic variability that allows species to evolve in response to environmental changes. Duplication represents a class of mutation that results in polymorphism in the copy number of genes. Investigating the phenotypic and fitness consequences of duplications is, however arduous, as it requires contemporary duplications under selection. Organophosphate and carbamate insecticides are used to control the main malaria vector, the mosquito *Anopheles gambiae*, and resistance to insecticides provides evolutionary biologists with ongoing adaptation to manmade modifications of the environment. Recent studies have revealed an intriguing complexity of resistance alleles at the *ace-1* locus (encoding the target of these insecticides), in particular different duplications involving various numbers and types (susceptible or resistant) of copies of this gene. We used an integrative approach, from genomic to phenotypic levels, to show that the various genomic architectures of these adaptive alleles result in different evolutionary trade-offs whose selection depends on insecticide distribution and intensity. However, this versatility also provides a high adaptability to *An*. *gambiae*, which may have a major impact on malaria vector control in Africa.

## Introduction

Gene duplications have long been considered to be rare (although some studies contradicted this perception, e.g. [1]), neutral events providing raw genetic material for long-term evolution. However, next-generation sequencing (NGS) technologies have revealed that copy-number variations (CNVs), such as deletions and duplications of genetic material, are widespread in natural populations (review in [2]). Increasing numbers of studies also suggest that CNVs may play a role in adaptation to environmental changes at the micro-evolutionary scale (for a review, [3]; see also [4,5]).

In homogeneous gene duplications (also referred to as gene amplifications in cases of successive repeats), the gene copies are identical. They can confer a quantitative advantage in situations in which having larger amounts of the corresponding protein is advantageous. Examples of such coding-gene duplications abound for proteins involved in functions directly related to the environment (e.g., resistance to xenobiotics through higher levels of detoxification, [6–8]; greater amylase production providing adaptation to a starch-rich diet in humans and dogs, [9,10]; higher hexose transporter levels for adaptation to an environment in which resources are limited, [11]). However, homogeneous duplications are also often associated with deleterious pleiotropic effects (or selective costs), probably due to the disruption of biochemical balance or overproduction costs ([12], e.g., in *Culex pipiens*, esterases may account for up to 12% of total protein, by weight, [13]).

By contrast, heterogeneous duplications comprise two different copies of the same gene; they can, thus, provide a more qualitative advantage, through the simultaneous production of two different proteins. It has been suggested that heterogeneous duplications are advantageous in contexts in which the heterozygote genotype is the fittest (i.e., overdominance). Heterogeneous duplications are not affected by the segregation burden carried by standard heterozygotes, and this allows the fixation of the heterozygote phenotype [14,15]. However, empirical evidence of heterogeneous duplications remains scarce, and the role of such duplications in adaptive processes is poorly documented. The few examples described to date concern genes targeted by insecticides: *rdl* in *Drosophila melanogaster* [16] and the parallel evolution of the *ace-1* locus in the West Nile mosquito *C*. *pipiens* and the malaria mosquito *An*. *gambiae*, in response to the use of organophosphate (OP) and carbamate (CX) insecticides [17–19].

OPs and CXs target acetylcholinesterase (AChE1), a synaptic enzyme encoded by *ace-1* in mosquitoes. The inhibition of this enzyme impairs hydroxylation of the neurotransmitter acetylcholine, inducing death through tetany [20]. Resistance is the consequence of a single-base substitution in the *ace-1* gene (*ace-1*^*R*^ allele, or R allele), resulting in an amino-acid substitution (G119S) in AChE1 that limits the insecticide binding [21]. This substitution has been selected in several mosquito species exposed to OPs and CXs [21–24]. However, in *Cx*. *pipiens* and *An*. *gambiae s*. *l*., the G119S substitution has also been shown to decrease the affinity of the resistant enzyme for its substrate by more than 60% relative to the susceptible version [25,26]. This lower affinity probably underlies the high selective cost of the R allele in both species [27–32].

Several heterogeneous duplications associating a susceptible S and a resistant R *ace-1* copy on the same chromosome (D alleles) have been described in natural populations of *Cx*. *pipiens* [17,18,33–35]. These duplications restore protein activity whilst maintaining substantial resistance, thus conferring a phenotype similar to that of a standard heterozygote (RS) [36]. A heterogeneous *ace-1* duplication has also recently been described in *An*. *gambiae s*. *l*. This duplication occurs in *Anopheles coluzzii* and *An*. *gambiae s*.*s*. and has spread over a large geographic area [19,37]. Its phenotypic consequences have been shown to be similar to those in *Cx*. *pipiens*: an intermediate level of resistance and a large decrease in the selective cost associated with the G119S mutation [32]. It has, therefore, been proposed that heterozygotes (RS) and individuals carrying heterogeneous *ace-1* duplications (DD, DS, or DR) may be selected in mosaics of treated and untreated areas, because they probably represent the best resistance/cost trade-off, outperforming cost-free SS susceptible or highly resistant but costly RR homozygotes [17,32,36,38].

Surprisingly, duplications involving several copies of an R allele per chromosome (hereafter R^x^, with x the number of copies) have also recently been reported in natural populations of *An*. *gambiae*, suggesting that relationships between *ace-1* CNVs and resistance to OPs and CXs may be more complex than previously thought [39,40]. However, the impact on fitness of these R^x^ duplications and the basis of their selection over single-copy alleles and/or heterogeneous duplications according to environmental conditions remain unclear.

Given the increasing use of OP and CX insecticides to control malaria mosquitoes, there is an urgent need to determine the roles of the various *ace-1* duplications (D and R^x^) in resistance. Furthermore, from an evolutionary point of view, this situation provides us with a rare opportunity to determine how the use of different genetic architectures enables an organism to cope with different environmental variations.

We present here an integrative study, from the genomic to the phenotypic level, of the role of *ace-1* duplications in *An*. *gambiae* resistance to OPs and CXs. We first determined the genomic structure of *ace-1* duplications, with base-resolution of the breakpoints. We found that the same 203 kb genomic region, encompassing the *ace-1* gene and 11 other genes, was amplified in all resistant mosquitoes, through heterogeneous (D) or homogeneous (R^x^) duplications. We then investigated the influence of the architecture of duplications and gene-dosage on mosquito fitness. We considered the implications of the results obtained in terms of both potential applications in resistance management, and fundamental evolutionary aspects such as adaptation to a changing environment.

## Results

### A Large Chromosomal Segment Encompassing *ace-1* Is Recurrently Duplicated in All Resistant *An*. *gambiae* Mosquitoes

#### *ace-1* is part of a 203 kb tandem heterogeneous duplication containing 12 genes

**Fig 1:**
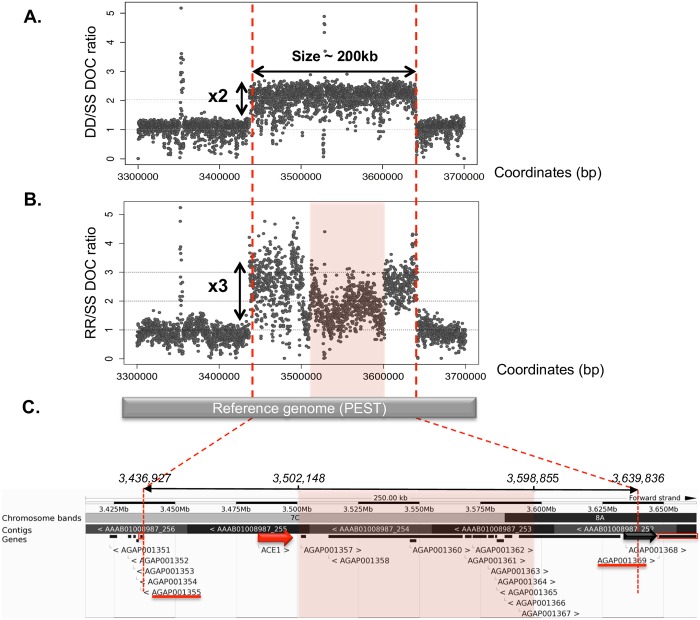
Genomic structure of the 202.91 kb amplicon encompassing the *ace-1* gene. (A) Acerduplikis strain (DD) unique segmental duplication event based on the DD/SS depth of coverage (DOC) ratio (SS corresponds to the KisumuP strain). (B) Acerkis strain (RR) multiple segmental duplication events based on the RR/SS DOC ratio. The pink box corresponds to a decrease in DOC by one third within the amplicon, suggesting that there has been a deletion in only one of the three copies. (C) Gbrowse view of the duplicated region in VectorBase (https://www.vectorbase.org). The duplicated region contains 12 genes: the *ace-1* locus (large red arrow) lies about 50 kb from its 5ʹ end, and a gene encoding a transposase overlaps its 3ʹ end (AGAP001368, large black arrow). Genes used as markers to delineate the amplicon are shown as red boxes, with their names underlined in red. Underlying data can be found in NCBI http://www.ncbi.nlm.nih.gov/bioproject/348825.

We characterized the genomic structure of the *ace-1* heterogeneous duplication (D allele, carrying one copy of the S allele and one copy of the R allele) by comparing the genomes of two strains, Acerduplikis (DD) and KisumuP (SS). Illumina-generated 250 bp spaced paired-end reads from both strains were first mapped onto the reference *An*. *gambiae* PEST genome (VectorBase; AgamP4; [41]). We then calculated the DD to SS ratio of the read depths of coverage (DOC; see Materials and Methods). We identified a clear 2-fold increase in coverage, of ~200 kb, on the 2R chromosomal arm encompassing the *ace-1* locus. This observation confirms the duplication of the *ace-1* locus but also reveals that this locus is part of a much larger duplicated fragment (Fig 1A).

This finding was further confirmed by assessing the insert size distribution for read pairs mapping close to the putative duplication breakpoints defined by the DOC ratio, for both the DD and SS strains. As expected, discordant pairs (*i*.*e*. with reads mapping ~200 kb apart) were found only in the DD strain (S1A Fig).

We investigated whether the two amplicons were in tandem by looking for soft-clipped reads, defined as reads encompassing the junction between amplicons, which would therefore map only partially onto the reference non-duplicated genome. As expected, soft-clipped reads were found only in the DD strain (S1B and S1C Fig). A multiple-sequence alignment of soft-clipped reads and discordant paired-end reads showed that the duplication was 202.91 kb in size (positions 3,436,927 to 3,639,836; Fig 1). This alignment also made it possible to reconstruct the sequences of the breakpoints and, thus, the sequence of the junction between the two amplicons (S1C Fig). The Illumina junction sequence was then confirmed by Sanger sequencing (S1D Fig), which revealed a strict tandem duplication event.

Finally, from the VectorBase reference genome, the duplication appeared to contain 12 genes (S1 Table), beginning with *ace-1* itself (~50 kb from the 5' breakpoint), whereas the AGAP001368 locus encoding the Harbinger transposase overlapped the 3' breakpoint (Fig 1C).

#### [RR] individuals, including those from the Acerkis reference strain, also display duplication

We used the junction sequence to develop a diagnostic PCR duplication test: each of the primers used (Agduplispedir2 and AgduplispeRev1, S1D Fig and S2 Table) binds to a different amplicon. This PCR amplifies a 460 bp fragment overlapping the junction in the Acerduplikis (DD) strain. It is therefore specific for individuals carrying the duplication. Ten *An*. *gambiae* field populations from Benin, Burkina Faso, Togo, and Ivory Coast (Table 1) were screened. All mosquitoes were first typed by *ace-1* PCR-RLFP, which distinguishes between the [SS], [RS], and [RR] phenotypes (it does not, however, distinguish between RS, DD, DS, and DR genotypes, which all provide [RS] phenotype) [22]. They were then tested with the diagnostic duplication test.

**Table 1:** Screening for the presence (+) or absence (-) of the *ace-1* duplication in West African *An*. *gambiae* populations.

Country	Site	Year	Species			Phenotype		
			*An*. *coluzzii*	*Hybrids*	*An*. *gambiae s*.*s*.	SS	RS	RR
Benin	Grand-Popo	2013	1	-	-	56 / **-**	7 / +	0
	Natitingou		-	-	1	54 / **-**	5 / +	1 / +
Burkina Faso	VK7	2011	1	-	-	18 / **-**	1 / +	0
	Boromo		0.48	-	0.52	17 / **-**	8 / +	0
	Dano		0.17	0.03	0.80	26 / **-**	4 / +	0
	Bobodioulasso		0.14	0.04	0.82	43 / **-**	3 / +	0
Togo	Baguida	2013	-	-	1	0	11 / +	52 / +
Ivory Coast	Tiassalé	2012	0.74	-	0.26	0	43 / +	0
	Toumodi		1	-	-	11 / **-**	0	0
	Bouaké		0.05	0.05	0.90	0	38 / +	0

Mosquitoes from various populations were first genotyped with the *ace-1* PCR-RLFP test [23]. The quick diagnostic PCR test for duplications, specific for the junction between duplicated copies (see text), was then applied to each individual. For each site, the following information is given: year of collection, proportion of individuals belonging to each species, number of individuals for each *ace-1* phenotype followed by the “-” or “+” symbols indicating the absence or presence of a duplication junction. Note that all [SS] mosquitoes gave negative results in the duplication test (225 individuals), whereas all [RS] or [RR] mosquitoes (173 individuals) gave positive results, regardless of the species considered. Underlying data can be found in DRYAD http://dx.doi.org/10.5061/dryad.4f7qg.

No amplicons were obtained in diagnostic duplication tests on [SS] individuals (Table 1), consistent with the presence of the S allele as a single copy in susceptible individuals. However, a 460 bp fragment similar to that detected in the DD strain was amplified from all [RS] individuals and, unexpectedly, from the 53 [RR] individuals from Baguida and Natitingou (Table 1). Even more surprisingly, this fragment was also amplified from the 20 individuals of the Acerkis [RR] resistant strain tested. Thus, all [RR] phenotypes in *An*. *gambiae* seem to have at least two R copies within tandem amplicons.

**Fig 2:**
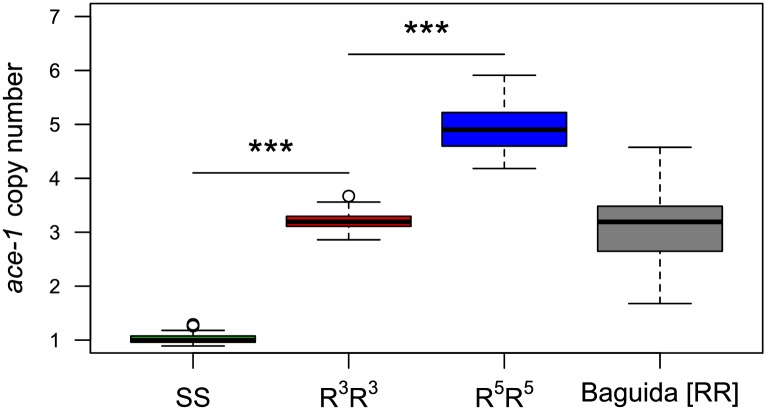
Number of *ace-1* copies in KisumuP, AcerkisR^3^, AgRR5 strains and in the Baguida [RR] population. Boxes indicate *ace-1* to *Rps7* gene concentration ratios (advanced relative quantification method, LightCycler 480 software 1.5.0). The significance of ratio differences between strains is indicated (***, *p* < 0.001). Underlying data can be found in DRYAD http://dx.doi.org/10.5061/dryad.4f7qg.

We estimated the number of *ace-1* copies in Acerkis [RR] mosquitoes by analyzing 32 mosquitoes from this strain by real-time quantitative PCR (qPCR). These mosquitoes had three times as many *ace-1* copies (3.21 ± 0.18) as individuals from the KisumuP (SS) strain (1.03 ± 0.09; LM, *t* = 61, *df* = 62, *p* < 0.001, Fig 2). They thus had three *ace-1* R copies per chromosome.

#### Similar structures for homogeneous R duplications and heterogeneous D duplications

Acerkis [RR] individuals were sequenced with Illumina technology (see Methods), for the precise characterization of their amplicons. We analyzed the breakpoints and junctions, as described above. As expected, the DOC RR/SS ratio around the *ace-1* locus was about three times that of the adjacent regions (Fig 1B).

Only one sequence was retrieved for the *ace-1* locus itself. These *ace-1*^*R*^ duplications were, thus, homogeneous: the same R copy was repeated. The allele carried by these Acerkis individuals consisted of three R copies, within amplicons organized strictly in tandem (as in the D allele). We will therefore refer to this allele as R^3^, and, as Acerkis individuals are homozygous, their genotype will hereafter be indicated by R^3^R^3^ and the strain used here renamed AcerKisR^3^ (the number of *ace-1* copies when the strain was isolated cannot be determined; S3 Table). Discordant (relative to the PEST reference genome) read pairs were found at exactly the same locations as for the D allele. Thus, all the amplicons, regardless of whether they carried the R (in R^3^ or D) or S (in D) allele, presented identical breakpoints (Fig 1).

However, the RR/SS DOC ratio was not uniform along the amplicons: an internal area not encompassing the *ace-1 locus*, in which this ratio was one third lower than elsewhere in the amplicon, suggested that a deletion had occurred in one of the three amplicons. Estimation on the basis of the discordant paired-end reads suggested that this deletion covered 97 kb (from 3,502,148–3,598,855, Fig 1B and 1C). Detailed analysis of the intra-amplicon deletion showed that it disrupted two genes (AGAP001357 and AGAP001367) and completely deleted eight others (AGAP001358; AGAP001360-1366); only *ace-1* remained complete in the amplicon with the deletion (the twelfth gene, AGAP001368, was disrupted during the amplification process, Fig 1C). Finally, a comparison of the Illumina amplicon sequences of R^3^R^3^ and DD individuals revealed the presence of several single-nucleotide polymorphisms (SNPs) at the 3ʹ-end of the DD amplicons, whereas the R^3^R^3^ amplicons were monomorphic at these positions. All these DD SNPs were a mixture of two bases, one of which was identical to that found in R^3^R^3^. Assuming that the polymorphism in DD was due to the presence of the S amplicon, we designed a pair of PCR primers (AgRDdir1 and AgRDrev1) to amplify a fragment containing this polymorphic region and the junction, to make it possible to determine the orientation of the duplication (S2 Table). The Sanger sequences of this fragment were strictly identical in R^3^R^3^ and DD individuals. Thus, in the D allele, the amplicon containing the R copy is positioned upstream from both the junction and the amplicon containing the S copy (S1B Fig).

#### Variable numbers of *ace-1* copies in the field

We used real-time qPCR to determine the number of *ace-1* copies in 39 [RR] mosquitoes from the Baguida (Togo) field population (Fig 2). There were 3.3 to 9.1 *ace-1* copies per individual. In a diploid, this corresponds to 1.67 to 4.57 copies per chromosome, with a median of about three R copies (median = 3.1, Fig 2). However, the distribution of copies per chromosome is unknown.

We investigated whether the *ace-1* amplicons segregating in field populations were similar to those identified in our strains, by quantifying, in individuals from Baguida (Togo) and from Tiassale and Bouake (Ivory Coast), the numbers of copies for two loci located just outside the amplified region (AGAP001355 and AGAP001369, referred to as 5'out and 3'out, respectively, see Methods, Fig 1C). All the individuals tested had only one copy of each of these genes, suggesting that their amplicons were similar in size to those of the DD and R^3^R^3^ strains.

### Fitness associated with homogeneous R duplications

The fitness impact of the D allele, the heterogeneous duplication pairing a susceptible, and a resistant copy of the *ace-1* gene has been assessed elsewhere [32]. The D allele was found to confer a lower level of resistance than the R allele, but it almost completely resorbed the high fitness cost associated with the G119S mutation for the various traits studied [32]. Surprisingly, we show here that the R allele used in this previous study (from the AcerkisR^3^ strain) is actually a homogeneous duplication of three copies of R. Consistent with other recent studies [39,40], we also found that the number of copies of R in [RR] individuals was variable in natural populations (Fig 2). We thus investigated the phenotypic and fitness consequences of different amplicon architectures and gene dosages.

#### AChE1R activity and insecticide resistance increase with the number of R copies

We first investigated the relationships between AChE1 activity and the number of R and S copies in various *ace-1* genotypes. We measured the activities of the resistant (A_R_) and susceptible (A_S_) forms of the enzyme in 40 mosquitoes (20 of each sex) of the KisumuP (SS), AcerkisR^3^ (R^3^R^3^), and Acerduplikis (DD) strains, and their F1 offspring (R^3^S, DS, and DR^3^, see S3 Table). AChE1 activities were consistently lower in females than in males (*t* = 3.7, *df* = 219, *p* < 0.001), so we analyzed the two sexes separately.

The AChE1 activity corresponding to one resistant copy (measured in R^3^R^3^ individuals as total activity divided by six) was one-quarter to one-fifth (0.21 ± 0.07 and 0.23 ± 0.07, for females and males, respectively) that of a single susceptible copy (measured in SS individuals), confirming the decrease in AChE1 activity associated with the G119S mutation [26]. In both sexes, highly significant correlations were found between A_S_ and the number of S copies in the genotype (Pearson’s correlation coefficient: *r* = 0.82, *t* = 16, *df* = 117, *p* < 0.001 and *r* = 0.84, *t* = 15, *df* = 96, *p* < 0.001, for females and males, respectively), and between A_R_ and the number of R copies in the genotype (*r* = 0.96, *t* = 37, *df* = 117, *p* < 0.001 and *r* = 0.93, *t* = 26, *df* = 96, *p* < 0.001, for females and males, respectively; S2 Fig).

**Fig 3:**
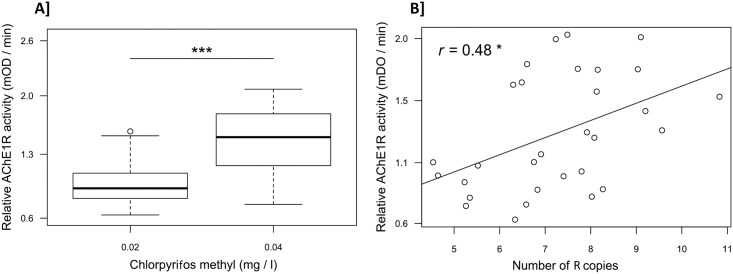
Relationship between AChE1 activity and insecticide resistance, or the number of R copies. (A) Boxplots present the distribution of AChE1R activity for [RR] individuals selected at low (0.02 mg/l, *n* = 30) and high (0.04 mg/l, *n* = 30) doses of chlorpyrifos methyl. ***: Student’s *t* test, *p* < 0.001. (B) Regression analysis showing a significant positive relationship (GLM, *: *p* < 0.05) between AChE1R activity and the number of R copies (as the distribution by chromosome is unknown, the total number of R copies is given for each individual). Underlying data can be found in DRYAD http://dx.doi.org/10.5061/dryad.4f7qg.

We assessed the relationships between *ace-1* R copy-number variations and mosquito AChE1R activity (Ar) on the one hand, and insecticide resistance on the other, by exposing a first batch of larvae from the Baguida field population to 2 x 10^−2^ mg/l chlorpyrifos methyl (leading to 30% mortality) and another batch of larvae to 4 x 10^−2^ mg/l (leading to 80% mortality). We analyzed 30 emerging adults from each batch and showed that AChE1R activity was significantly higher (1.5 ± 0.4 times) in larvae surviving exposure to 4 x 10^−2^ mg/l chlorpyrifos methyl than in larvae surviving 2 x 10^−2^ mg/l (Student’s *t* test: *t* = -5.8, *df* = 53, *p* < 0.001, Fig 3A). We then measured the number of R copies (Nc) in 30 [RR] survivors (15 from each batch) for which AChE1R activity had previously been measured. There was a significant positive correlation between individual AChE1R activity and the number of R copies (GLM: Ar = Nc + ε, with ε the error parameter (Gaussian distribution), *t* = 2.7, *df* = 28, *p* = 0.01, Fig 3B).

More precise investigations of the effects of the number of R copies on fitness required a strain carrying a homogeneous duplicated allele with more R copies than R^3^ (AcerkisR^3^). Baguida larvae were selected by exposure to 2 x 10^−2^ mg/l chlorpyrifos methyl. Surviving females were crossed with SS males and allowed to lay eggs individually. We used the PCR-RFLP test [22] to phenotype these females for the *ace-1* locus, to identify [RR] females. We determined the number of *ace-1* copies in these females by qPCR. For females carrying more *ace-1* copies than AcerkisR^3^ individuals, we determined the number of *ace-1* copies in six second-instar larvae from their progenies. We found identical copy numbers in all six larvae (i.e., indicating that the mother was homozygous) in only one of the 70 progenies tested. These larvae all had six *ace-1* copies; as they were [RS], they therefore carried five *ace-1* copies on the same chromosome (i.e., an R^5^ allele). The rest of this progeny was backcrossed five times successively with SS males (KisumuP) to homogenize the genetic background. This strain thus had a genetic background similar to those of KisumuP (SS) and AcerkisR^3^ (R^3^R^3^). Mosquitoes were then crossed with each other, to fix the homozygous resistant phenotype [RR], to generate the AgRR5 strain. Quantitative PCR was used to confirm that the individuals of this strain were all of genotype R^5^R^5^ (*n* = 30 individuals, 4.93 ± 0.42 R copies per chromosome, Fig 2).

A comparison between the two [RR] strains showed that R^5^R^5^ individuals had AChE1R activity levels 1.5 ± 0.1 times those of R^3^R^3^, close to the expected value assuming strict additivity according to the number of copies present (1.7, S3 Fig).

Bioassays were then carried out on larvae from the three strains (SS, R^3^R^3^ and R^5^R^5^), with different insecticides: one CX (bendiocarb), one OP (chlorpyrifos methyl) and one pyrethroid (PYR, permethrin). Mortality in the controls never exceeded 5%. SS, R^3^R^3^ (RR_50_ = 0.97, *p* > 0.05), and R^5^R^5^ (RR_50_ = 0.99, *p* > 0.05) mosquitoes were all susceptible to permethrin, confirming that the *kdr* alleles present in the Baguida field population had been eliminated during the backcrosses (Table 2). For the two insecticides targeting AChE1, bendiocarb and chlorpyriphos-methyl, the R^5^R^5^ strain displayed significantly higher resistance than R^3^R^3^ (RR_50_ = 290 versus 207, *p* < 0.001 and RR_50_ = 14 versus 12, *p* < 0.001, respectively) (Table 2 and S4 Fig).

**Table 2:** Dose-mortality responses to various insecticides observed in the various *Anopheles gambiae s*. *s*. strains.

	Insecticide								
Genotype	Bendiocarb			Chlorpyrifos-methyl			Permethrin		
	LC_50_	RR_50_	Chi(*p*)	LC_50_	RR_50_	Chi(*p*)	LC_50_	RR_50_	Chi(*p*)
SS (KisumuP)	0.27	-	a	0.003	-	a	0.005	-	a
R^3^R^3^ (AcerkisR^3^)	56.5	207	b	0.032	12	b	0.005	0.97	a
R^5^R^5^ (AgRR5)	79.1	290	c	0.037	14	c	0.005	0.99	a

LC_50_: Concentration (mg.l^-1^) inducing 50% mortality. RR_50_: Resistance ratio = LC_50_ (Strain) / LC_50_ (KisumuP). Chi(*p*): Comparison of the resistance levels conferred by the various genotypes; strains with different letters are significantly different. Underlying data can be found in DRYAD http://dx.doi.org/10.5061/dryad.4f7qg.

#### Increasing the number of R copies increases selective costs

We assessed the fitness cost associated with the different *ace-1* genotypes by comparing several life history traits between KisumuP (SS), AcerkisR^3^ (R^3^R^3^), and AgRR5 (R^5^R^5^).

**Fig 4:**
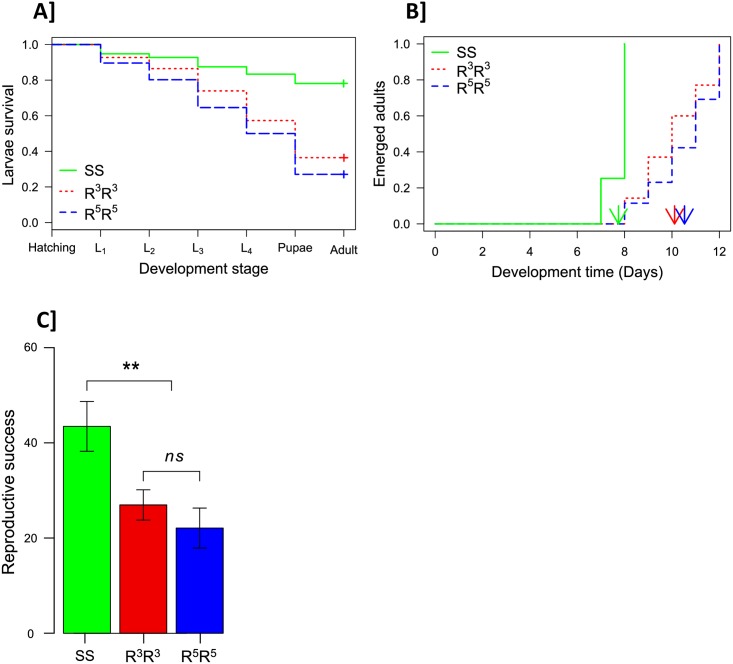
Life history traits for the KisumuP (SS), AcerkisR^3^ (R^3^R^3^) and AgRR5 (R^5^R^5^) strains. (A) *Larval mortality*: the proportion of larvae surviving at each development stage is presented, from hatching to emergence (L_i_ is the larval stage *i*); "+" indicates the proportion of emerged adults. (B) *Development time*: the proportion of emerged adults on each day after the start of the experiment is presented for each genotype; arrows indicate the mean development time of each genotype. (C) *Female fecundity*: the mean number of larvae per female in each strain is presented with its standard error; the significance of differences in fertility is indicated (n.s., *p* > 0.05; **, *p* < 0.01). Underlying data can be found in DRYAD http://dx.doi.org/10.5061/dryad.4f7qg.

We assessed pre-imaginal mortality from egg hatching to adult emergence. We recorded the number of dead larvae at each developmental stage, to assess differences between the strains in overall mortality and mortality dynamics. Overall mortality was highest for R^5^R^5^ individuals (*mR*^*5*^*R*^*5*^ = 0.73 [0.63–0.82]; the 95% confidence intervals are given in brackets), followed by R^3^R^3^ (*mR*^*3*^*R*^*3*^ = 0.64 [0.53–0.73]), with much higher survival rates for SS (*mSS* = 0.22 [0.15–0.32]). The SS genotype had a significantly different mortality pattern (Cox model: SS versus R^3^R^3^, *z* = 5.3, *p* < 0.001; SS versus R^5^R^5^, *z* = 6.4, *p* < 0.001), with a lower mortality at each larval stage, the patterns being similar for the R^3^R^3^ and R^5^R^5^ genotypes (Cox model: *z* = 1.5, *p* = 0.14; Fig 4A).

Development time was recorded as the number of days required for a first-instar larva to reach adulthood (i.e., the time until emergence). We detected no interaction between sex and genotype (GLM, likelihood ratio test [LRT]: *χ*^*2*^ = 2.64, *Δdf* = 2, *p* = 0.26) and no sex effect (8.8 ± 1.5 d for males and 8.98 ± 1.7 d for females; LRT: *χ*^*2*^ = 0.4, *Δdf* = 1, *p* = 0.55). However, R^3^R^3^ and R^5^R^5^ individuals had similar development times (10.11 ± 1.38 and 10.53 ± 1.36 days, respectively; Cox model: *z* = -0.9, *p* = 0.38), with both developing significantly more slowly than SS individuals (7.74 ± 0.43 days; Cox model: *z* = -7.78, *p* < 0.001 and *z* = -8, *p* < 0.001; Fig 4B).

We assessed the influence of R copy-number variation on female reproductive success by allowing 40 females of each genotype (SS, R^3^R^3^ and R^5^R^5^) to lay eggs. Overall reproductive success did not differ significantly between R^3^R^3^ and R^5^R^5^ females (mean ± SE = 27 ± 3.2 and 22 ± 4.3 larvae per female, respectively; GLM: *F* = 0.64, *Δdf* = 1, *p* = 0.43) but was significantly lower for both these genotypes than for SS females (43 ± 5.2 larvae per female; GLM: *F* = 6.8, *Δdf* = 2, *p* < 0.01; Fig 4C). The observed differences were due solely to R^3^R^3^ and R^5^R^5^ females laying fewer eggs than SS females (GLM: *F* = 12.2, *Δdf* = 2, *p* < 0.001; S5A Fig), as neither the proportion of females laying eggs nor the hatching rate per female differed significantly between the three genotypes (GLM: *χ*^*2*^ = 1.5, *Δdf* = 2, *p* = 0.47, S5B Fig, and *F* = 0.93, *Δdf* = 2, *p* = 0.40, S5C Fig, respectively).

Overall, the performance of R^5^R^5^ mosquitoes did not differ significantly from those of R^3^R^3^ mosquitoes for any of the development, mortality, or fecundity traits analyzed. Nevertheless, the mean performances of R^5^R^5^ mosquitoes were always slightly lower than those of R^3^R^3^ mosquitoes for all these traits (Fig 4), suggesting that R^5^R^5^ individuals may actually be subjected to slightly higher costs than R^3^R^3^ individuals.

We carried out an experimental evolution study, taking the whole life cycle into account, to confirm this trend. Competition between the R^3^ and R^5^ alleles was established by crossing 250 females of the AcerkisR^3^ strain (R^3^R^3^) with 250 males from the AgRR5 strain (R^5^R^5^). Their F1 progeny (all R^3^R^5^) was reared in standard conditions (27 ± 2°C, 80 ± 2% humidity and 12h:12h light/dark cycle), in the absence of insecticide. After emergence, the adults were released into a new cage and allowed to reproduce freely for five discrete generations. Three replicates (C_1_, C_2_ and C_3_) were set up.

Total AChE1 activity, which is correlated with *ace-1* copy number (S3 Fig), was used to assess the change in the proportions of the R^3^ and R^5^ alleles in the cages. For each replicate, we measured total AChE1 activity for 32 individuals from each generation: two measurements were made per individual, to limit measurement error, and only females were analyzed to avoid a sex effect. Five individuals each of the two reference strains, AcerkisR^3^ (R^3^R^3^) and AgRR5 (R^5^R^5^), were used as controls. For each individual, an activity index (A_I_) was constructed as follows: A_I_ = (A_x_ - A_R3_) / (A_R5_ - A_R3_), where A_x_ is the mean total activity of individual x, and A_R3_ and A_R5_ are the mean total activities estimated for the control genotypes. An A_I_ close to 0 corresponds to an AChE1 activity similar to that of R^3^R^3^ individuals, whereas a value close to 1 corresponds to an AChE1 activity similar to that of R^5^R^5^ individuals.

We followed the change in mean A_I_ index in each replicate across the five discrete generations. We used the following GLM to determine whether A_I_ changed over generations: A_I_ = Gen + ε, where Gen is a five-level factor corresponding to the generations and ε is a Gaussian error parameter. In all replicates, the mean activity index (A_I_) decreased significantly, from 0.53 ± 0.07 to 0.23 ± 0.10, between G1 and G5 (GLM: *t* = -2.13, *df* = 163, *p* < 0.05; *t* = -6.21, *df* = 163, *p* < 0.001 and *t* = -3.15, *df* = 163, *p* < 0.01; for C_1_, C_2_, and C_3_, respectively). This result suggests that the R^5^ allele tends to be eliminated by R^3^ (S6 Fig), consistent with the trend previously observed for individual life history traits.

## Discussion

Recent NGS studies have revealed that CNVs are pervasive in natural populations. Altering the number of copies of a gene is generally thought to be deleterious, although some CNVs have been shown to be adaptive. For example, contemporary duplications of the *ace-1* gene underlying resistance to organophosphate (OP) and carbamate (CX) insecticides have been observed in several mosquito species (*Cx*. *pipiens*, *An*. *gambiae*, and *An*. *albimanus*, [18,37,42]), in one moth species (*Plutella xylostella*, [43]), and in two spider-mite species (*Tetranychus urticae* and *Tetranychus evansi*, [44,45]). The number of *ace-1* copies is variable (up to five in *T*. *urticae* and *T*. *evansi*), but duplications are usually heterogeneous, involving at least one susceptible copy (S) and one resistant copy (R). *An*. *gambiae* is unusual in that both heterogeneous and homogeneous (with only R copies) duplications have been selected independently and segregate in natural populations ([19,39], this study).

### *ace-1* Gene Duplications Are Pervasive in *An*. *gambiae* Populations

By resolving the genomic structure of these duplications, we were able to design a diagnostic test for duplications that revealed that *ace-1* was systematically duplicated in resistant mosquitoes, but never in susceptible mosquitoes (there were 173 resistant individuals among the 398 mosquitoes from four sub-Saharan African countries tested). The 200-kb amplicon is also found in specimens from Burkina Faso and Guinea sequenced by the *An*. *gambiae* 1,000 Genomes Consortium (manuscript in preparation) and occurs only in specimens carrying the resistance allele. This confirms that *ace-1* duplications are adaptive, whether heterogeneous (with one S and one R copy) or homogeneous (with multiple R copies), and pervasive in natural populations of *An*. *gambiae*.

Genomic analysis also revealed that the *ace-1* locus was part of the same 203 kb amplicon in both kinds of duplications. Amplicons are arranged strictly in tandem (i.e., contiguous to each other), with identical breakpoints and junctions, down to individual base level. Heterogeneous duplications contained only one S copy and one R copy, whereas the number of R copies was more variable in homogeneous duplications (up to five copies were detected).

OPs and CXs were introduced relatively recently (over the last 50 y) for the control of malaria vector populations. The number and diversity of the duplicated alleles suggest that these duplications are relatively common events. This conclusion is consistent with recent studies showing that the rate of gene duplication per gene and per generation ranges from a value similar to the substitution rate to a value four orders of magnitude higher [46–49]. It has been suggested that such duplications are promoted by the presence of repeated elements [50,51], which favor unequal crossing-over events by inducing chromatin mismatching. Indeed, several studies have reported the presence of transposable elements at the breakpoints of large adaptive segmental duplications [4,16,52]. We also identified a transposable element (the Harbinger transposase) at the 3ʹ end of the amplicons. The non-allelic homologous recombination model (NAHR [53]) is, thus, probably the most parsimonious mechanism explaining this high frequency of duplication events with conserved breakpoints around the *ace-1* amplicons.

### All Resistance Alleles Share the Same 203 kb Amplicon, Precluding Specific Genotyping

It was not possible to design a PCR-based molecular test specific for the *ace-1* R copy to differentiate between heterogeneous and homozygous duplications, as the R^x^ and D alleles shared the same R copy, identical to the only R copy described to date [19,21].

Genomic structure was also conserved among homogeneous and heterogeneous duplications, precluding the development of specific DNA markers to distinguish between amplicons containing R^x^ and D alleles on the basis of breakpoints or junction sequences. The diagnostic duplication test developed in this study can thus be used only to determine whether a mosquito carries a duplication of some kind. Furthermore, while the amplicons carrying the S or R copy in the D allele had several diagnostic SNPs at their 3' ends, they were identical at their 5ʹ ends, as the amplicon carrying the R copy was located upstream from that carrying the S copy, making it impossible to develop a PCR test specific for the R-S association.

Finally, while it is possible to evaluate the number of *ace-1* copies with high precision by qPCR, there is currently no method for determining the distribution of copies between homologous chromosomes. Thus, if qPCR and PCR-RFLP *ace-1* tests on a particular [RS] individual indicate the presence of four *ace-1* copies, for example, it is not possible to determine whether the genotype of this individual is DD, DR^2^, or R^3^S. Indirect methods, such as coupling molecular tests with extensive crossing experiments, thus remain the only way to determine the precise genotype, and such methods are unsuitable for use in large field screenings.

In our study, we used such indirect methods to genotype the offspring of individuals crossed with the reference SS strain. The quantification of *ace-1* copy number by this approach was precise enough to determine the exact genotype when one of the chromosomes was known (here, the chromosome carrying S). This made it possible to establish strains with various numbers of R copies, to investigate the effect of *ace-1* CNVs on the phenotype and fitness of mosquitoes.

### Different Genomic Architectures Result in Different Fitness Trade-Offs

We previously investigated the phenotypic and fitness impacts of heterogeneous duplications of *ace-1* in *An*. *gambiae* [32]. We showed that the D allele confers a phenotype resembling that of standard (RS) heterozygotes, with an intermediate level of resistance and a lower fitness cost. In this study, we showed that acetylcholinesterase activity, regardless of the form of the enzyme (susceptible [AChE1S] or resistant [AChE1R]), is globally proportional to the number of S or R copies, respectively, in the genotype (S2 Fig). Consequently, the D allele has a higher level of activity than a single R copy, because the G119S mutation greatly decreases AChE1 activity. The partial restoration of AChE1 activity induced by the combination of R and S on a single chromosome probably accounts for the lower fitness cost and the intermediate level of resistance of the D allele [32]. The D allele thus provides a new and intermediate alternative to the otherwise irreducible trade-off between protein activity and resistance level (S: no cost, no resistance; R: high cost, high resistance).

By contrast to heterogeneous duplications, which combine qualitatively different alleles, homogeneous duplications usually provide a quantitative advantage through repeats of the same allele (see the review by [3]). In the case of *ace-1*, the total AChE1 activity provided by R^x^ alleles appears to be positively correlated with the number of R copies (i.e., the number of amplicons). However, no quantitative advantage in terms of resistance was anticipated; resistance was thought to be a binary trait conditioned solely by target protein conformation [21]. The finding that resistance level increased with the number of R copies (R^5^R^5^ individuals clearly displayed higher resistance than R^3^R^3^ individuals, S4 Fig) was therefore surprising, indicating that resistance is more quantitative than was previously thought (Fig 3).

However, the relationship between cost and copy number may not be monotonic. First, life history traits and experimental evolution studies comparing R^5^ and R^3^ alleles suggested that a larger number of R copies was associated with a higher cost (Fig 4 and S6 Fig). Conversely, this relationship may not hold if there are fewer than three R copies: (i) three R copies per chromosome were found in the AcerkisR^3^ strain [RR], despite the absence of insecticide selection over a number of generations (at least 4 y; if carrying fewer copies was less costly, this number would be expected to decrease with time in the absence of selection); (ii) the median number of copies in the Baguida field population (Togo) was three, although only a few [RR] individuals with different numbers of copies were found. It thus seems that three R copies on the same chromosome may be optimal in terms of the associated cost, with carrying fewer or more copies entailing higher fitness costs.

**Fig 5:**
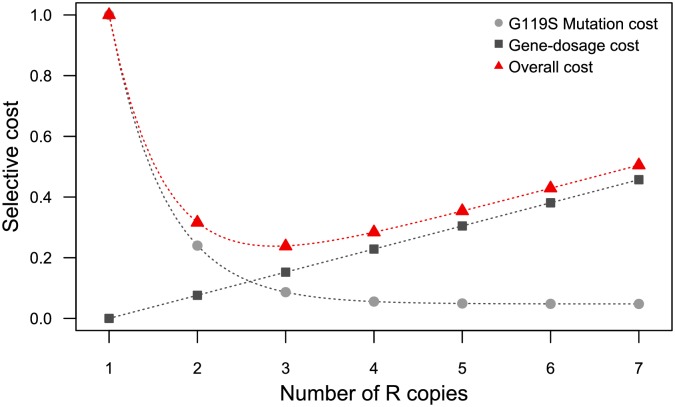
Hypothetical model to explain the non-monotonous relationship between fitness cost and the number of R copies, and the minimum cost centered on three copies per chromosome. Under this hypothesis, the overall fitness cost (red triangles) is the result of two opposite functions: the cost associated with G119S mutation (light gray circles) and the cost due to the change in gene dosage (dark gray squares). The first of these costs is negatively correlated with the number of R copies. Thus, larger numbers of R copies are associated with lower costs. By contrast, the second cost is positively correlated with the number of R copies. Note that the shapes of each function and their interaction are unknown.

So how can we explain this surprising situation? Where do these costs come from? It is possible that total cost is a combination of at least two different, opposite functions correlated with amplification level (Fig 5). First, the cost associated with the low level of AChE1R activity (cost of the G119S mutation) probably decreases with increasing numbers of R copies (e.g., the number of amplicons). The paucity of individuals with only one- or two-copy alleles in the field suggests a high cost; conversely, the total activity provided by the R^5^ allele is higher than that of the R^3^ allele, but less than that of the susceptible allele (no overshoot cost). The second cost function would increase with the number of amplicons: R^5^ appears to be more costly overall than R^3^. As the two alleles have the same amplicon and structure, this cost is probably metabolic rather than structural in nature. First, as AChE1 is involved in numerous functions at different stages of development (for a review see [54]), the production of larger amounts of AChE1 may have a deleterious effect on these other functions (i.e., a pleiotropic effect). The second possibility, which is probably more likely, is that this cost is not directly related to *ace-1* itself, instead being due to changes in the dosage of one or several of the other genes encompassed by the duplication (i.e., gene dosage cost, Fig 5).

The two functions are probably not symmetric, as the activity of the R^5^ allele was similar to the expected value under an additive model, whereas the increase in cost relative to R^3^ was barely visible in terms of life history traits. This hypothetical combination of two cost functions is consistent with the observations and suggests that the R^3^ allele may be optimal (Fig 5).

### Internal Deletions May Reduce the Gene-Dosage Disruption Cost

Genome analyses revealed that the 203-kb amplicon contained 12 genes, only one of which (*ace-1*) is involved in resistance to OPs and CXs (S2 Table). Amplicon size may be constrained by the presence of the Harbinger element, favoring the occurrence of large duplications encompassing the other 11 loci. However, these genes are probably only hitchhiking, so their duplication is unlikely to be adaptive. By contrast to *ace-1*, an increase in their gene-dosage may actually be deleterious: (i) it could alter biochemical equilibria between the duplicated genes and the single-copy genes with which they interact [55,56]; (ii) the function of these duplicated genes may be disrupted because optimal levels of protein may be overshot [57,58]; or (iii) the superfluous production of excess protein from the duplicated genes may be energetically costly [59]. These three hypotheses are not mutually exclusive.

We report here observations supporting the notion that duplication costs are due, at least in part, to gene-dosage imbalance between the co-amplified genes rather than directly to *ace-1*. In R^3^R^3^ individuals (AcerKis), DOC decreased within some of the amplicons (Fig 1B), revealing 97 kb intra-amplicon deletions affecting the 11 loci other than *ace-1*. In these individuals, only *ace-1* remains fully amplified, the number of copies of the other genes having decreased towards their initial dosage (Fig 1B). Moreover, this internal deletion was recurrent and also detected in mosquitoes from Burkina Faso and Guinea sequenced by the *An*. *gambiae* 1,000 Genomes Consortium (manuscript in preparation).

These observations suggest that the intra-amplicon deletions occurred after the duplication events. More importantly, they suggest that these secondary deletions are probably adaptive and were selected to reduce the cost of dosage imbalance for the 11 genes concerned. These deletions would alter the shape of the gene-dosage cost function (Fig 5). This process is probably still ongoing, and it should be possible to test this adaptive hypothesis by comparing fitness between individuals carrying the same number of R copies with and without these deletions.

### The Genomic Architecture Selected for Resistance Alleles Probably Depends on the Intensity of Selective Pressure, and This Must Be Taken into Account in Vector Control

The key finding of this study is that the various architectures of the *ace-1* amplicon correspond to different evolutionary trade-offs between resistance and costs, and that they all segregate in *An*. *gambiae* field populations. The reason for this coexistence of different alleles with different fitness trade-offs probably lies in the diversity of treatment practices for vector control. This diversity often results in mosaic environments, composed of patches covering the whole gradient from untreated to intensely treated areas, leading to the selection of different alleles. Moderate treatments or fine-grained alternation of treated and untreated areas (over time and/or space) should favor the heterozygous phenotype, with moderate resistance and costs (overdominance and marginal overdominance, respectively). As the D allele does not bear the segregation burden of standard heterozygotes, this heterogeneous duplication should, thus, be favored in this context [17,32,36]. This may be the case in *An*. *gambiae* populations from the Ivory Coast, which contained only [RS] individuals, all displaying amplification (Tiassalé and Bouaké, *n* = 43 and 38, respectively), suggesting that D is present at high frequency, if not already fixed (Table 1). By contrast, higher insecticide doses and coarse-grained environments should select more specialist phenotypes, and, thus, homogeneous duplications with higher levels of resistance (despite their higher costs). Moreover, higher doses should select for larger numbers of copies (R^5^ confers stronger resistance than R^3^). In this respect, variations in the level of amplification in homogeneous duplications should allow a more finely tuned response to variations in selection intensity, as the quantitative effects of such duplications on fitness seem to be more subtle than those of heterogeneous duplication.

The demonstration of this versatility in the trade-offs available through duplication represents a major development in our understanding of the evolutionary processes of adaptation. However, the consequences, in terms of vector control, are much more negative. Following the massive increase in PYR insecticide resistance in African populations of *An*. *gambiae*, several African countries have tried to preserve the efficacy of vector control by switching from PYRs to CXs or OPs, in accordance with the American President’s Malaria Initiative [60], in collaboration with the National Malaria Control Program [61]. However, duplicated *ace-1* resistance alleles are already widespread. Some populations contained only resistant individuals, with either 100% [RS] individuals (suggesting the presence of the D allele, Tiassalé and Bouaké, Ivory Coast) or with mostly [RR] individuals (83% carrying R^X^ alleles, Baguida, Togo).

Very careful management of the doses of insecticide used, and of their spatial and temporal application, will be required to control resistance, as treatment could rapidly lead to the selection of different types of *ace-1* alleles, hampering mosquito control. Such fine-tuning of treatment may prove difficult in a context in which insecticides are used for vector control and various compounds used in agriculture can also select *ace-1* resistance alleles (for a review [62]). This issue is particularly pressing because OPs and CXs have been recommended in the context of a high frequency of PYR resistance alleles, but recent studies have reported alarming synergic effects between resistance alleles specific for each insecticide class [63,64].

The high adaptability conferred by *ace-1* duplications may have a major impact on *An*. *gambiae* vector control in Africa, potentially impeding the control of malaria transmission.

## Materials and Methods

### Mosquito Strains and Collections

#### Mosquito strains

Three *An*. *gambiae* laboratory strains that were already available were used in this study: KisumuP, Acerkis, and Acerduplikis. KisumuP was derived from the susceptible reference strain Kisumu [65] and was rendered homozygous for a single susceptible *ace-1*^*S*^ allele (or S, genotype SS; [32]). Acerkis and Acerduplikis are resistant to both OPs and CXs [32,66]. Acerkis is homozygous for the G119S mutation in the *ace-1* gene (*ace-1*^*R*^ allele or R, genotype RR). Acerduplikis is homozygous for the *ace-1* heterogeneous duplicated allele (*ace-1*^*D*^ allele or D, genotype DD), associating one R copy and one S copy on the same chromosome. The three strains mostly share the same KisumuP genetic background (>99.6% similarity [32]).

#### Mosquito collection

Larvae from ten *An*. *gambiae* field populations were collected and reared until adulthood in the laboratory: two from Benin, four from Burkina Faso, one from Togo, and three from Ivory Coast (Table 1). Adults were assigned to members of the *An*. *gambiae* cryptic-species complex on the basis of morphological tests and molecular analyses [67,68]; their *ace-1* phenotype (susceptible [SS], homozygous resistant [RR], or heterozygous [RS]) was assessed through the *ace-1* PCR-RLFP test [22].

### Genomic DNA Preparation and Sequencing

Genomic DNA was extracted from individual mosquitoes of the three strains with the Qiagen DNeasy kit and was treated with RNase A to remove residual RNA. DNA concentration was assessed in the Qbit dsDNA BR Assay (LifeTechnologies). Illumina whole-genome sequencing libraries were constructed with the Nextera DNA sample preparation kit (Illumina), in accordance with the manufacturer’s instructions.

KisumuP (SS, two individuals) and Acerduplikis (DD, eight individuals) samples were sequenced by the Wellcome Trust Sanger Institute, as part of the *Malaria* genome project (https://www.malariagen.net/projects/vector/ag1000g). FASTQ format libraries were generated from 100 bp-read pairs separated by a 250 bp insert. These reads were mapped to the *An*. *gambiae* PEST reference genome assembly, downloaded from VectorBase (https://www.vectorbase.org; AgamP4; [41]) with a Wellcome Trust pipeline including Picard tools (https://broadinstitute.github.io/picard/) to format the data, bwa (0.7.5a-r405 [69]) for mapping, and other analysis tools providing optimized mapping data in SAM format.

The Acerkis (RR, two individuals) samples were specifically sequenced for this project with the same sequencing technology (Illumina). The sequencing data consisted of 125 bp-read pairs separated by a 500 bp insert. The reads were then trimmed and mapped directly, using bwa, onto the same PEST reference genome.

For the genomic analysis, we used the gene annotations from the AgamP4.3 (https://www.vectorbase.org/organisms/anopheles-gambiae/pest/AgamP4.3).

### Duplication Detection and Characterization

We used a two-step approach to detect and characterize the structure of the duplications containing the *ace-1* locus. We first analyzed the variations in read depth of coverage (DOC) for short-read mappings on the PEST reference genome. This made it possible to detect CNVs; duplications induce a local increase in DOC, whereas deletions induce a local decrease in DOC. We specifically focused on the genomic region in which the *ace-1* locus is located, from the 2 Mb to 5 Mb positions on chromosomal arm 2R (AgamP4.3 release; https://www.vectorbase.org/organisms/anopheles-gambiae/pest/AgamP4.3; Fig 1). Rather than calculating the DOC for each base, we determined the number of reads falling into adjacent 100 bp windows. This made it possible to minimize the computer resources required without losing DOC information. We detected DOC shifts, by calculating the DOC ratio for each resistant strain (i.e., the DOC of the considered strain, RR, or DD, over the DOC of the SS strain, which has no *ace-1* gene duplication Fig 1A and 1B).

We merged all the sequencing data for individuals of the same strain to intensify the signal and its resolution. This made it possible to confirm and refine the location of the duplication by resolving its breakpoints. In particular, we analyzed the insert size distribution of the paired-end reads (S1A Fig) close to the previously defined putative breakpoints (± 1 kb; S1B Fig). Breakpoint sequences can be determined precisely by analyzing soft-clipped reads (i.e., partially mapped reads indicative of tandem duplication events as they overlap two copies) and discordant read pairs (i.e., pairs with a mapping span and/or orientation inconsistent with the expected insert size). The soft-clipped reads and discordant pairs were selected by parsing the mapping data in SAM format https://samtools.gihub.io/hts-spes/SAMv1.pdf. We then aligned them with MUSCLE, a multiple alignment tool suitable for short sequences available through SeaView alignment editor/viewer version 4 [70,71]. The consensus sequences of both the breakpoints (i.e., 5ʹ and 3ʹ flanking sequences of the duplication) and the junction (i.e., sequences overlapping the two amplicons; S1C Fig) were then constructed.

We determined the 5'–3' orientations and relative positions of the different amplicons by amplifying a 2,465 bp PCR fragment (AgRDdir1 and AgRDrev1 primers) overlapping the junction from the genomic DNA of individuals (S1 Table). PCR products were purified with the QIAquick Gel Extraction Kit (Qiagen) and directly sequenced with an ABI Prism 310 sequencer (BigDye Terminator Kit, Applied Biosystems, Foster City, CA).

### Quick Diagnostic PCR Test for Duplications

A PCR primer pair was designed (Agduplispedir2 and AgduplispeRev1), with each primer binding to a different amplicon, on either side of the junction (S1D Fig and S1 Table). The resulting 460 bp fragment overlaps the junction, and is therefore amplified only in individuals carrying multiple copies of the *ace-1* locus. This quick, simple diagnostic PCR test thus reveals the presence or absence of *ace-1* duplications.

### Gene Copy-Number Quantification

We estimated the number of copies present for several target loci relative to a reference locus AGAP010592) present as a single copy in the VectorBase PEST genome (*Rps7*, AgS7Ex5qtidir and AgS7Ex5qtirev primers; https://www.vectorbase.org/, S1 Table), by real-time quantitative PCR (qPCR, LC480 Light Cycler, Roche). Three genes were targeted: *ace-1* (AGAP001356, AgAce1qtidir2, and AgAce1qtirev2 primers) and two loci flanking the amplified region: AGAP001355 on the 5ʹ-flanking side ("5'out", Ag5ʹoutdir and Ag5ʹoutrev primers), and AGAP001369 on the 3ʹ-flanking side ("3'out", Ag3ʹoutdir and Ag3ʹoutrev primers; Fig 1 and S1 Table). These flanking loci were used as qPCR markers of the amplified zone. As they are located outside the amplicon, they should not be amplified.

We dispensed 0.5 μl of genomic DNA and 1.5 μl of reaction mixture containing specific primers, each at a concentration of 0.8 μM and 0.75 μl of Master Mix (LightCycler 480 SYBR Green I Master, Roche) into the wells of a 384-well plate, with a Labcyte Echo525 dispenser. We performed qPCR as follows: activation at 95°C for 8 min, followed by 45 cycles of 95°C for 4 s, 67°C for 13 s, and 72°C for 19 s. Melting curves were generated by a post-amplification melting step between 70°C and 95°C, for Tm analysis. All quantifications were replicated four times for each DNA template. Standard curves were constructed with 10-fold dilutions of a PCR product previously amplified with specific primers for each locus from KisumuP (SS) strain DNA. The *ace-1*, 5ʹout, and 3'out concentration ratios over *RpS7* were determined by the advanced relative quantification method (LightCycler 480 software v.1.5.0).

### Resistance Measure

Bioassays were used to assess mosquito resistance to three insecticides: one CX (bendiocarb, technical grade, 99.5% purity), one OP (chlorpyrifos methyl, 99.9% purity), and one PYR (permethrin, 98.3% purity). We incubated 25 late third-instar larvae for 24 h at 27°C ± 2°C in plastic cups containing 99 ml of distilled water, to which we added 1 ml of insecticide solution at the required concentration (1 ml of ethanol in controls). Four replicates were performed for each concentration. Larval mortality was recorded after 24 h of exposure. Dose-mortality responses were analyzed with the BioRssay R script (v.6.2 [72]) freely available from the ISEM website (http://www.isem.univ-montp2.fr/recherche/teams/genomic-adaptation/staff/labbepierrick/?lang=en). This script computes the 50% or 95% lethal concentrations (LC_50_ and LC_95_, i.e., insecticide doses killing 50% and 95% of the tested population or strain) and their confidence intervals, and assesses the linearity of the dose-mortality response (*χ*^*2*^ test). Finally, it compares the dose-mortality responses of two or more strains (or populations) and calculates their resistance ratios (RR_50_ or RR_95_, = LC_50_ or LC_95_ of the tested strain/population over LC_50_ or LC_95_ of the reference strain, respectively) and 95% confidence intervals.

### AChE1 Activity Measure

Adult mosquitoes were decapitated, and each head was individually homogenized in 400 μl phosphate buffer (0.25 M, pH7) supplemented with 1% Triton X-100. Homogenates were centrifuged (9.3 g for 3 min) and 100 μl of the supernatant was dispensed into each of two wells of a 96-well microtitration plate. We added 10 μl ethanol (95%) to the first well and 10 μl propoxur (a CX insecticide, at 10^-1^M, diluted in ethanol) to the second. The plate was incubated for 15 min at room temperature. We then added 100 μl of substrate solution (25 mM sodium phosphate, pH 7.0, 0.2 mM DTNB, 0.35 mM sodium bicarbonate, 2.5 mM acetylthiocholine) to each well. AChE1 activity was estimated by measuring the change in optical density following the cleavage of acetylthiocholine, as described by Ellman et al. [73]. Optical density at 412 nm was recorded every minute for 15 min with an EL 800 microplate reader (Bio-Tek Instruments, Inc.). The mean slope of each reaction was calculated with KCjunior v1.41.4 analysis software (Bio-Tek Instruments, Inc.) and was used as a measurement of AChE1 activity.

The first well (ethanol) was used to assess total AChE1 activity (A_TOT_), whereas the second (propoxur) provided information about AChE1R activity (A_R_) only. AChE1S activity (A_S_) was deduced as = A_TOT_—A_R_. Note that activity in the second well was never equal to 0: a very shallow slope was observed even for susceptible individuals, due to the spontaneous degradation of DTNB. For comparisons of different strains or populations, samples of each were distributed on the same plate and analyzed simultaneously to avoid experimental artifacts.

### Proxies for Fitness Cost

#### Larval mortality and development time

We assessed the development time and pre-imaginal mortality associated with different *ace-1* copy numbers by performing larval mortality assays on different strains, as described by Assogba et al. [32]. Briefly, first-instar larvae were individually reared in *Drosophila* tubes, in 1 ml of mineral water with TetraMin powdered fish food (2 g/l). Dead larvae were counted daily to assess the mortality rate at each development stage. The timing of adult emergence was also recorded.

#### Female fecundity and fertility

All strains were reared under the same soft environmental conditions (relatively low densities, no food limitation). In each strain, 200 males were crossed with 200 females. Females were blood-fed after 3 d, and 40 gravid females from each strain were allowed to oviposit individually in plastic cups containing 70 ml dechlorinated water. Three days after blood feeding, the number of egg-laying females and the number of eggs per female were recorded. Two days later, we determined the number of hatching larvae per female.

### Statistical Analyses

#### *ace-1* copy-number variation

The numbers of *ace-1* copies for individuals from different strains were estimated by qPCR. The significance of the differences observed was assessed with the following generalized linear model (GLM): Cn = Geno + *ε*, where Cn is the number of copies, Geno is a multi-level factor corresponding to genotype and *ε* is the error parameter, which follows a Gaussian distribution. We checked the normality of the model residuals in a Shapiro-Wilk test [74].

#### Fitness costs

Larval mortality was analyzed by calculating the following Cox proportional hazards regression model (Cox model): Surv = Geno + *ε*, where Surv is the proportion of dead larvae at each developmental stage, Geno is a three-level factor corresponding to the different genotypes tested, and ε is the error parameter, which follows a binomial distribution to take any overdispersion into account. Emerging adults were censored in the analyses.

Differences in development time between genotypes and/or sexes were assessed with the following Cox model: Dev = Geno + Sex + Geno.Sex+ *ε*, where Dev is the number of adults emerging on a given day, Geno is a three-level factor corresponding to the different genotypes tested, Sex is a two-level factor (male or female), Geno.Sex is the interaction between these two factors, and ε is the error parameter (binomial distribution).

The other cost proxies (Cp) were analyzed with GLMs in the form Cp = Geno + *ε*, where Geno is a three-level factor corresponding to the different genotypes tested and *ε* is the error parameter, which follows a binomial distribution for the proportion of females laying eggs and the hatching rate, and a Gaussian distribution for the numbers of eggs and larvae per female.

All calculations were performed with free R software (v.3.1.1, http://www.r-project.org). LM, GLM, and Cox models were simplified as follows: the significance of the different terms was assessed, beginning with the higher-order terms, in likelihood ratio tests (LRTs), and non-significant terms (*p* > 0.05) were removed. The factor levels of qualitative variables that were not significantly different (in LRT) were grouped [75].

### Data Availability

Data deposited in the Dryad repository: http://dx.doi.org/10.5061/dryad.4f7qg [76]. Sequence data deposited in the NCBI repository: http://www.ncbi.nlm.nih.gov/bioproject/348825.

## Supporting Information

S1 Fig Resolution of the *ace-1* duplication structure. **(A) Distribution of the paired-end (PE) insert size in the vicinity of the breakpoints (± 1 kb)**. For each strain, we recorded the insert size of each read and its paired read; for each 200 bp insert size class, we calculated the number of reads, which was then normalized relative to the 2R chromosome mean DOC (between 2 Mb and 5 Mb, excluding the duplicated region). Discordant PEs presented insert sizes distributed around 202 kb, and were identified only for the Acerduplikis (DD, mean insert size 250 bp) and AcerkisR^3^ (R^3^R^3^, mean insert size 500 bp) strains. **(B) Duplication breakpoints and junction resolution**. The top figure shows the relative positions of the amplicons (*i*.*e*. carrying the resistant R or susceptible S copy) of the DD strain duplication; the 5’ end of the amplified region is crosshatched in blue and the 3’ end is crosshatched in red. The bottom figure shows the expected mapping of the reads from DD strain onto the reference genomes: PE reads surrounding the duplication junction result in discordant pairs (*i*.*e*. pairs with reads mapping in opposite orientations, with an insert size different from the expected 250 bp); reads overlapping the duplication junction result in soft-clipped reads (*i*.*e*. partially mapped reads). These features were used to estimate the duplication length and to reconstitute the junction and breakpoint sequences. (**C) Alignment of breakpoints and junction sequences**. The 5’ and 3’ breakpoint sequences are aligned with the junction sequence. (**D) Junction sequences for the Acerduplikis (DD) and AcerkisR**^3^ **(R**^**3**^**R**^**3**^**) strains**. The two sequences are strictly identical; the junction position is indicated in the red box. The Agduplispedir2 and Agduplisperev1 primers used for sequencing and for the diagnostic test for duplications are highlighted in gray (see S2 Table). Underlying data can be found in DRYAD http://dx.doi.org/10.5061/dryad.4f7qg.
(PDF)

S2 Fig Relative AChE1 activities of the various genotypes. Relative AChE1R activities (scaled by the mean AChE1R activity of the R^3^R^3^genotype, top panels) and relative AChE1S activities (scaled by the mean AChE1S activity of the SS genotype, bottom panels) are shown for various genotypes, as a function of their number of R or S *ace-1* copies. The linear regression is plotted as a dotted line. Underlying data can be found in DRYAD http://dx.doi.org/10.5061/dryad.4f7qg.
(PDF)

S3 Fig Relative AChE1R activity in R^3^R^3^ and R^5^R^5^ individuals. Boxplots representing the relative AChE1R activity distribution measured on 20 males from the AcerkisR^3^(R^3^R^3^, red) and AgRR5 (R^5^R^5^, blue) strains. Differences in activity were assessed with the following GLM: Activity = Geno + ε, where Geno is a two-level factor corresponding to the genotype and ε is the error parameter, which follows a Gaussian distribution (***, *p* < 0.001). Underlying data can be found in DRYAD http://dx.doi.org/10.5061/dryad.4f7qg.
(PDF)

S4 Fig Resistance to bendiocarb (CX) and chlorpyrifos-methyl (OP) insecticides. Mortality (probit scale) is presented as a function of insecticide dose (log_10_) for the three strains: KisumuP (SS; green squares), AcerkisR^3^(R^3^R^3^, red triangles) and AgRR5 (R^5^R^5^, blue dots). Linear regressions between the two factors (solid lines) are indicated, together with the associated 95% confidence intervals (dotted lines). Underlying data can be found in DRYAD http://dx.doi.org/10.5061/dryad.4f7qg.
(PDF)

S5 Fig Female fertility and fecundity in susceptible (SS) and resistant (R^3^R^3^and R^5^R^5^) homozygotes. For each genotype, SS (green), R^3^R^3^ (red) and R^5^R^5^ (blue), we present the following: (A) the mean oviposition rate (*i*.*e*. the number of females laying eggs over the number of females studied) and its standard error (SEM), (B) the mean number of eggs laid per female and its SEM, and (C) the mean hatching rate (*i*.*e*. the number of larvae produced over the number of eggs) and its SEM. The significance of the differences between the various genotypes is indicated (n.s., *p* < 0.05; ***, *p* < 0.001). Underlying data can be found in DRYAD http://dx.doi.org/10.5061/dryad.4f7qg.
(PDF)

S6 Fig Dynamics of AChE1 activity index (A_I_) over generations in the experimental evolution assay. For each replicate (C_1_, C_2_ and C_3_), boxplots represent the distribution of activity index (A_I_) for each generation. Blue and red lines correspond to the expected A_I_ of R^5^R^5^ and R^3^R^3^ homozygotes, respectively. For each replicate, the green line corresponds to the following GLM: A_I_ = Gen + ε, where Gen is a five-level factor corresponding to generation and ε is the error parameter, which follows a Gaussian distribution. Underlying data can be found in DRYAD http://dx.doi.org/10.5061/dryad.4f7qg.
(PDF)

S1 Table List of the 12 genes present within the duplicated region and their function (from VectorBase AgamP4 *Anopheles gambiae* genome). (PDF)

S2 Table List of the primers used in this study. (PDF)

S3 Table Nature and number of *ace-1* copies in different mosquito genotypes. (PDF)

## Abbreviations

AChE1: acetylcholinesterase
CNV: copy-number variation
CX: carbamate
DOC: depth of coverage
NAHR: non-allelic homologous recombination
NGS: next-generation sequencing
OP: organophosphate
PYR: pyrethroid
