# Supplementary material for: The ace-1 Locus Is Amplified in All Resistant Anopheles gambiae Mosquitoes: Fitness Consequences of Homogeneous and Heterogeneous Duplications
Source: PLoS Biol. 2016 Dec 5;14(12):e2000618. doi: 10.1371/journal.pbio.2000618 (PMC5137868; doi:10.1371/journal.pbio.2000618)
Supplement: S1 Fig — (A) Distribution of the paired-end (PE) insert size in the vicinity of the breakpoints (± 1 kb). For each strain, we recorded the insert size of each read and its paired read; for each 200 bp insert size class, we calculated the number of reads, which was then normalized relative to the 2R chromosome mean DOC (between 2 Mb and 5 Mb, excluding the duplicated region). Discordant PEs presented insert sizes distributed around 202 kb, and were identified only for the Acerduplikis (DD, mean insert size 250 bp) and AcerkisR3 (R3R3, mean insert size 500 bp) strains. (B) Duplication breakpoints and junction resolution. The top figure shows the relative positions of the amplicons (i.e. carrying the resistant R or susceptible S copy) of the DD strain duplication; the 5’ end of the amplified region is crosshatched in blue and the 3’ end is crosshatched in red. The bottom figure shows the expected mapping of the reads from DD strain onto the reference genomes: PE reads surrounding the duplication junction result in discordant pairs (i.e. pairs with reads mapping in opposite orientations, with an insert size different from the expected 250 bp); reads overlapping the duplication junction result in soft-clipped reads (i.e. partially mapped reads). These features were used to estimate the duplication length and to reconstitute the junction and breakpoint sequences. (C) Alignment of breakpoints and junction sequences. The 5’ and 3’ breakpoint sequences are aligned with the junction sequence. (D) Junction sequences for the Acerduplikis (DD) and AcerkisR3 (R3R3) strains. The two sequences are strictly identical; the junction position is indicated in the red box. The Agduplispedir2 and Agduplisperev1 primers used for sequencing and for the diagnostic test for duplications are highlighted in gray (see S2 Table). Underlying data can be found in DRYAD http://dx.doi.org/10.5061/dryad.4f7qg. (PDF) [file pbio.2000618.s001.pdf]

A.

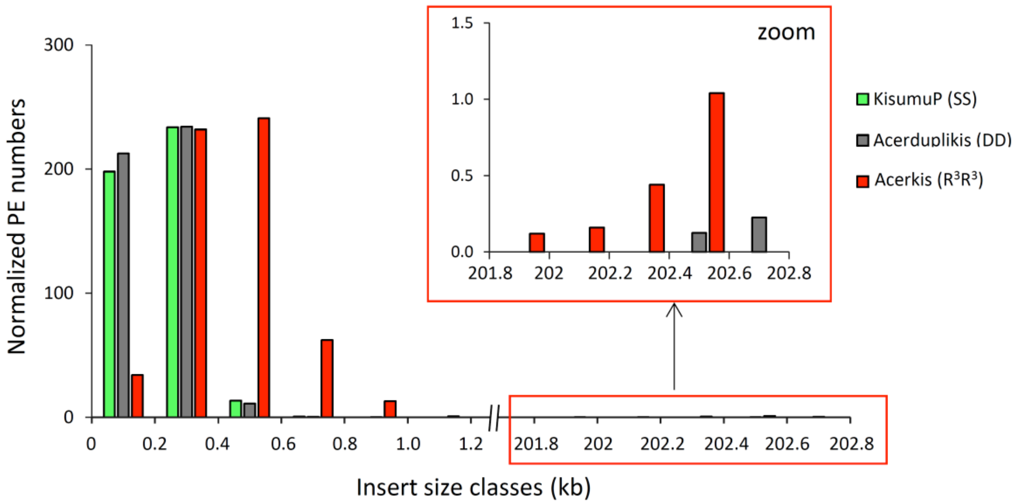

B.

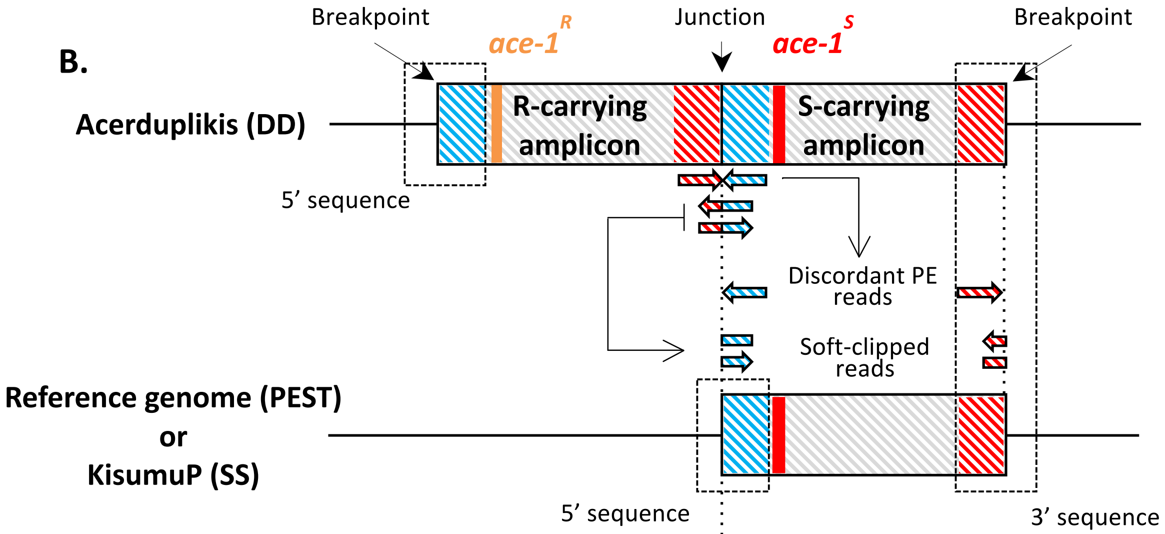

C.

```
5' sequence  TCTTATCATGTGCTTGGTGACAATTTTGTTTTTC-GAATCCACGGAGCGGGTGCGATTTAGGGCAGGACCGAT
   Junction  ATATTTTCCCCATTCTATTTTATCCGACGAAGCG-GAATCCACGGAGCGGGTGCGATTTAGGGCAGGACCGAT
3' sequence  ATATTTTCCCCATTCTATTTTATCCGACGAAGC--AAACCTGCAAAGTGGAAACAATAAGCTCTATCTGCAAC
```

D.

```
             1    Agduplispedir2
Acerduplikis CTCTTAAGGTGGCGTTGTTCCACCCCTTCTTCAAACAAACAATGCTCCAACAATAGGCGGTTGTTATGATACTGAAGCATATTTTCCCCATTCTATTTTA
Acerkis      ....................................................................................................
Acerduplikis TCCGACGAAGCGGAATCCACGGAGCGGGTGCGATTTAGGGCAGGACCGATGCTCAGTTTAGCGAGGGTGGCAGTGATGGTGGAATCGAGTCGAGATAAGC
Acerkis      ....................................................................................................
Acerduplikis TGGACGAGATTAACCCGAGCTCTTTTGGTAAAGCTTGGCTCTCGTGTCGCACCTGGTCAACTGGACAATCGATTCGATTTGGCTACCGATTGTAAAGCGT
Acerkis      ....................................................................................................
Acerduplikis GTGCACCTGGGTTACCGATATCAAAGCAGACGGGCCCACCAAGACAAGCAATAGCGTCAGCGTGCTGTGCTGCTACCTAAAGCGTAGTGTGTTTTAGACA
Acerkis      ....................................................................................................
Acerduplikis GAAAAGCAAGAAAAGCATAGGAGTTTTTGTTGCCGCATCTTTGCCAACCTTTTGTGCGAA
Acerkis      ............................................................
                                                      Agduplisperev1   460
```
