# Supplementary figures and images for: The ace-1 Locus Is Amplified in All Resistant Anopheles gambiae Mosquitoes: Fitness Consequences of Homogeneous and Heterogeneous Duplications
Source: PLoS Biol. 2016 Dec 5;14(12):e2000618. doi: 10.1371/journal.pbio.2000618 (PMC5137868; doi:10.1371/journal.pbio.2000618)

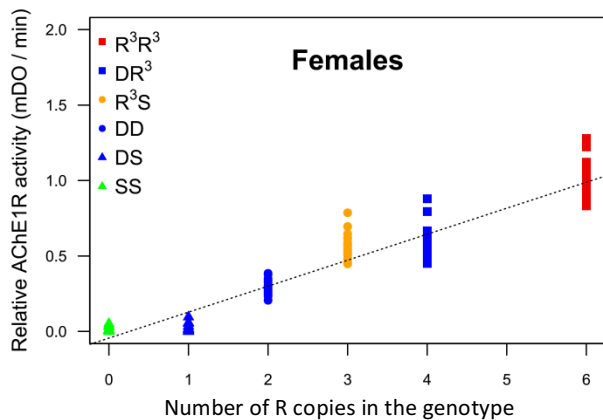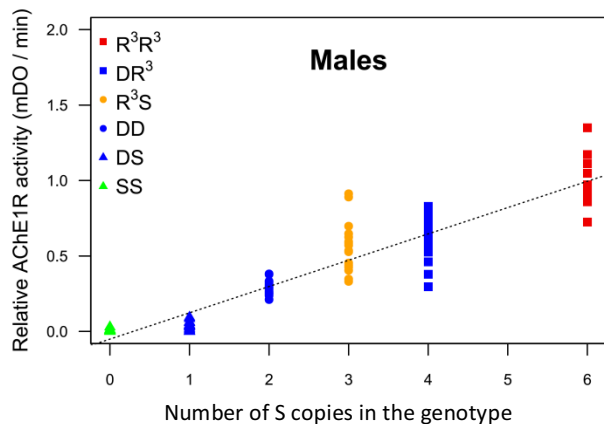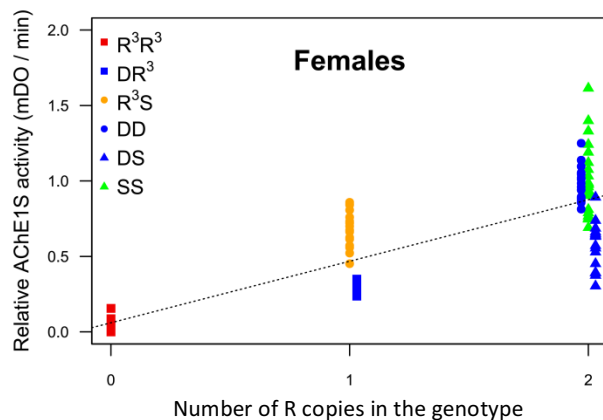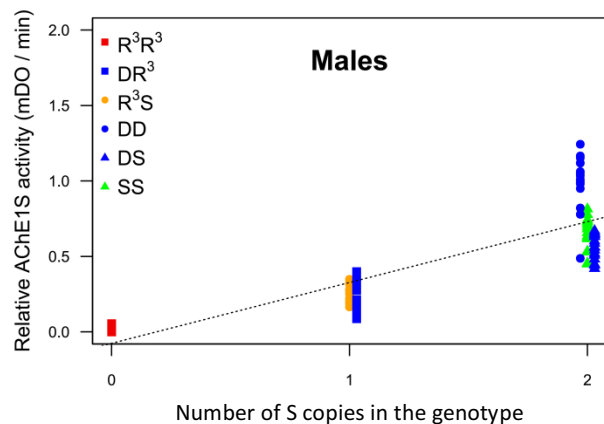

Supplement: S2 Fig — Relative AChE1R activities (scaled by the mean AChE1R activity of the R3R3genotype, top panels) and relative AChE1S activities (scaled by the mean AChE1S activity of the SS genotype, bottom panels) are shown for various genotypes, as a function of their number of R or S ace-1 copies. The linear regression is plotted as a dotted line. Underlying data can be found in DRYAD http://dx.doi.org/10.5061/dryad.4f7qg. (PDF) [file pbio.2000618.s002.pdf]

Relative AChE1R activity

1.8  
1.6  
1.4  
1.2  
1.0  
0.8  
0.6

\*\*\*

$R^3R^3$

$R^5R^5$

8

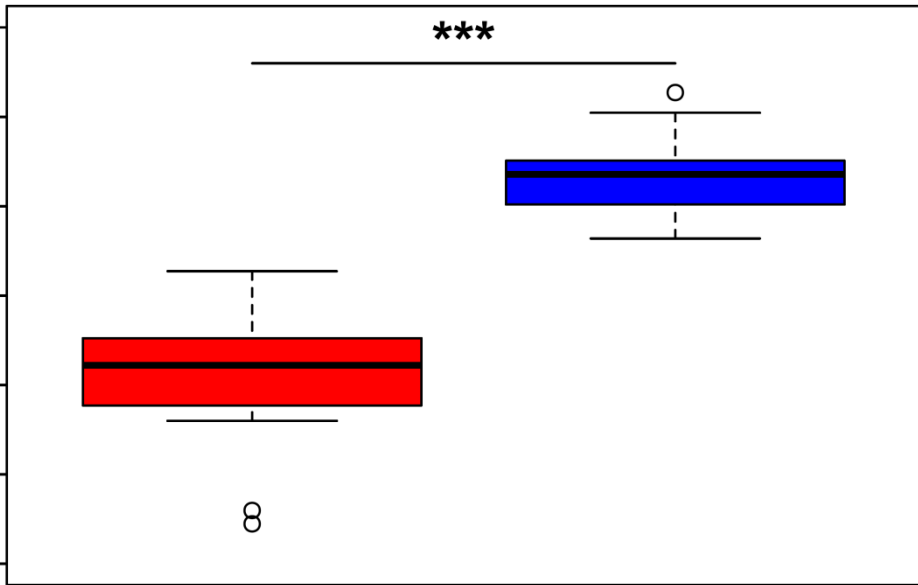

Supplement: S3 Fig — Boxplots representing the relative AChE1R activity distribution measured on 20 males from the AcerkisR3(R3R3, red) and AgRR5 (R5R5, blue) strains. Differences in activity were assessed with the following GLM: Activity = Geno + ε, where Geno is a two-level factor corresponding to the genotype and ε is the error parameter, which follows a Gaussian distribution (***, p < 0.001). Underlying data can be found in DRYAD http://dx.doi.org/10.5061/dryad.4f7qg. (PDF) [file pbio.2000618.s003.pdf]

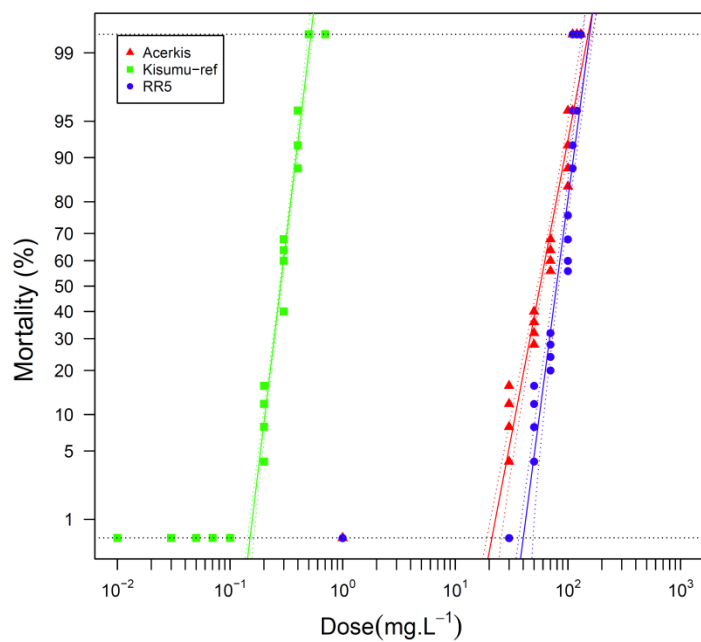

SS

R<sup>3</sup>R<sup>3</sup>

R<sup>5</sup>R<sup>5</sup>

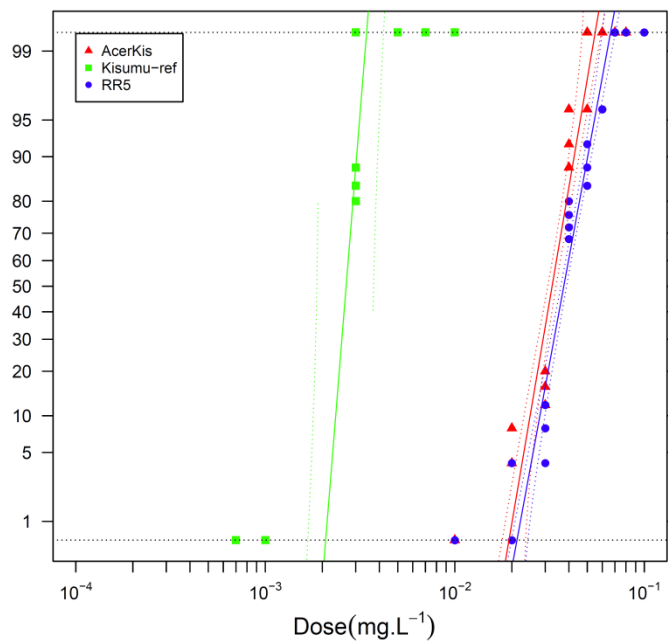

Supplement: S4 Fig — Mortality (probit scale) is presented as a function of insecticide dose (log10) for the three strains: KisumuP (SS; green squares), AcerkisR3(R3R3, red triangles) and AgRR5 (R5R5, blue dots). Linear regressions between the two factors (solid lines) are indicated, together with the associated 95% confidence intervals (dotted lines). Underlying data can be found in DRYAD http://dx.doi.org/10.5061/dryad.4f7qg. (PDF) [file pbio.2000618.s004.pdf]

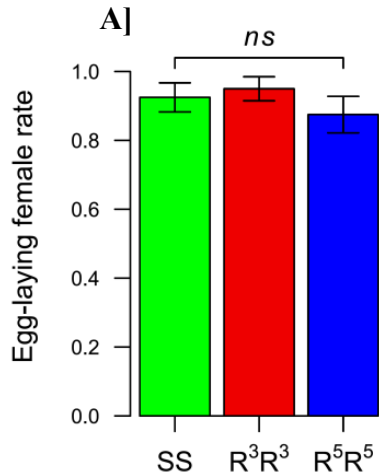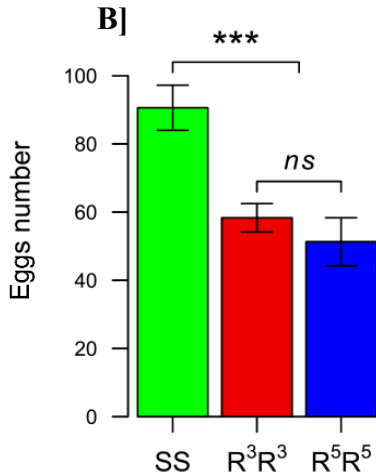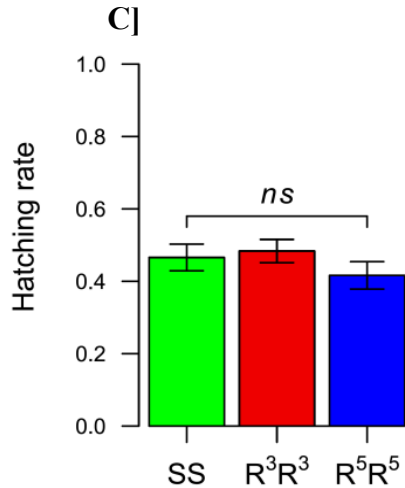

Supplement: S5 Fig — For each genotype, SS (green), R3R3 (red) and R5R5 (blue), we present the following: (A) the mean oviposition rate (i.e. the number of females laying eggs over the number of females studied) and its standard error (SEM), (B) the mean number of eggs laid per female and its SEM, and (C) the mean hatching rate (i.e. the number of larvae produced over the number of eggs) and its SEM. The significance of the differences between the various genotypes is indicated (n.s., p < 0.05; ***, p < 0.001). Underlying data can be found in DRYAD http://dx.doi.org/10.5061/dryad.4f7qg. (PDF) [file pbio.2000618.s005.pdf]

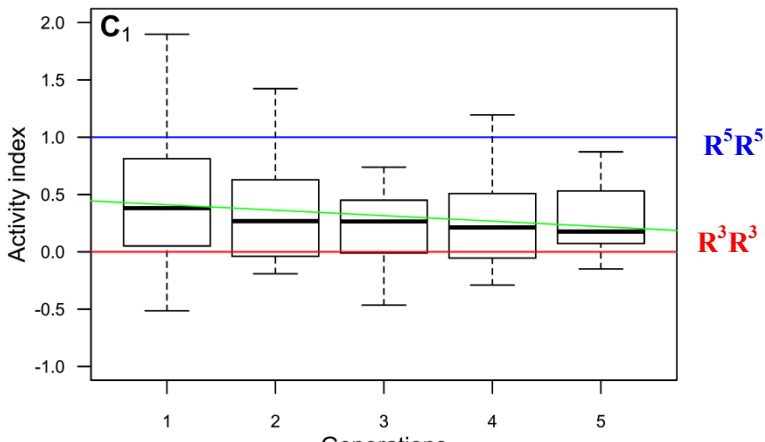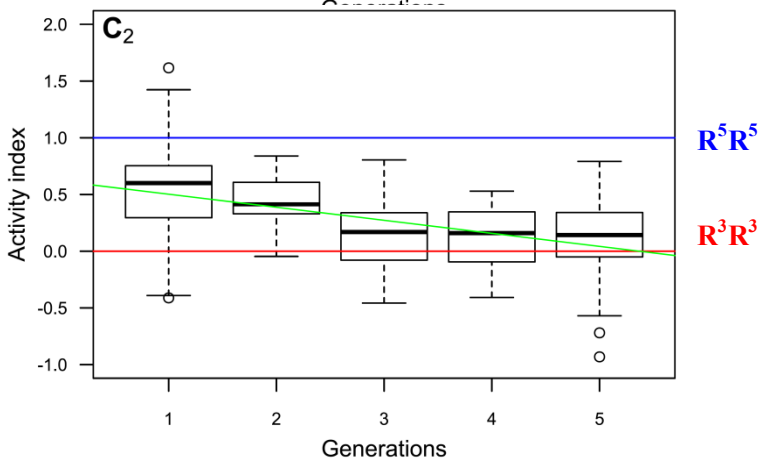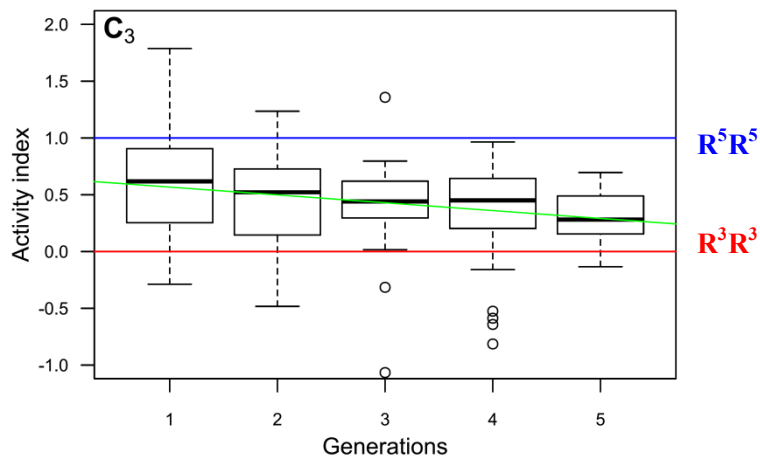

Supplement: S6 Fig — For each replicate (C1, C2 and C3), boxplots represent the distribution of activity index (AI) for each generation. Blue and red lines correspond to the expected AI of R5R5 and R3R3 homozygotes, respectively. For each replicate, the green line corresponds to the following GLM: AI = Gen + ε, where Gen is a five-level factor corresponding to generation and ε is the error parameter, which follows a Gaussian distribution. Underlying data can be found in DRYAD http://dx.doi.org/10.5061/dryad.4f7qg. (PDF) [file pbio.2000618.s006.pdf]
