# Supplementary material for: The ace-1 Locus Is Amplified in All Resistant Anopheles gambiae Mosquitoes: Fitness Consequences of Homogeneous and Heterogeneous Duplications
Source: PLoS Biol. 2016 Dec 5;14(12):e2000618. doi: 10.1371/journal.pbio.2000618 (PMC5137868; doi:10.1371/journal.pbio.2000618)
Supplement: S1 Table — (PDF) [file pbio.2000618.s007.pdf]

S1 Table: List of the 12 genes present within the duplicated region and their function (from VectorBase AgamP4 *Anopheles gambiae* genome).

locus	Chromosome	Genomic position	Protein coding	Protein function
AGAP001356	2R	3,484,107-3,495,790	*ace-1*	Neurotransmitter catabolic process
AGAP001357	2R	3,501,596-3,503,538	Catenin-beta-like (CTNNBL)	Role in apoptosis
AGAP001358	2R	3,512,832-3,567,719	Zinc finger-Ring C3HC4	Zinc ion binding
AGAP001360	2R	3,546,131-3,548,750	Zf-Ran BP	Zinc ion binding
AGAP001361	2R	3,568,692-3,571,356	DUF3337	Domain of unknown function
AGAP001362	2R	3,571,835-3,577,510	WD40-repeat proteins	Signal transduction and transcription regulation
AGAP001363	2R	3,578,169-3,580,460	Cohesin loading	Maintenance of mitotic sister chromatid cohesion
AGAP001364	2R	3,580,984-3,581,797	Ubiquitin fold modifier 1	Ubiquitin-like modifier protein
AGAP001365	2R	3,581,705-3,584,603	Trypsin	Serine-type endopeptidase activity
AGAP001366	2R	3,584,942-3,588,013	Trypsin	Serine-type endopeptidase activity
AGAP001367	2R	3,590,764-3,632,244	Glycosyltransferase family 43	galactosylgalactosylxylosylprotein, 3-beta-glucuronosyltransferase activity
AGAP001368	2R	3,634,518-3,645,862	DDE superfamily endonuclease	DNA transposition
