# Supplementary material for: The ace-1 Locus Is Amplified in All Resistant Anopheles gambiae Mosquitoes: Fitness Consequences of Homogeneous and Heterogeneous Duplications
Source: PLoS Biol. 2016 Dec 5;14(12):e2000618. doi: 10.1371/journal.pbio.2000618 (PMC5137868; doi:10.1371/journal.pbio.2000618)
Supplement: S2 Table — (PDF) [file pbio.2000618.s008.pdf]

S2 Table: List of the primers used in this study.

Marker name	Locus	Chromosome	Primer (5'-3')	Size (bp)	Tm (°C)
Diagnostic duplication test	AGAP001368 [AGAP001355- AGAP001356]	2R	Agduplispedir2: CTCTTAAGGTGGCGTTGTTCC<br>AgduplispeRev1:TTCGCACAAAAGGTTGGCA	460	60
Duplication orientation	AGAP001368 [AGAP001355- AGAP001356]	2R	AgRDdir1: ATGACCGTCCGTAGAGTGCT<br>AgRDrev1: TATTGCTTGTCTTGGTGGGC	2465	52
qPCR	AGAP001356 (*ace-1*)	2R	AgAce1qtidir2 : ATGTGGAACCCGAACACG<br>AgAce1qtirev2 : ACCACGATCACGTTCTCCTC	185	67
	AGAP010592 (*RpS7*, control)	3L	AgS7Ex5qtidir : GTGTACAAGAAGCTGACTGGC<br>AgS7Ex5qtirev : TAGCTGCTGCAAACTTCGG	107	67
	AGAP001355	2R	Ag5'outdir : CTCGAGAGCAACATTCAGGG<br>Ag5'outrev : GGAAGTTACGCAGCACCG	100	67
	AGAP001369	2R	Ag3'outdir : AGAACTCGAAAAACACGCCC<br>Ag3'outrev : GTCATCGCGTTCTTCAGGC	118	67
