# Supplementary material for: The ace-1 Locus Is Amplified in All Resistant Anopheles gambiae Mosquitoes: Fitness Consequences of Homogeneous and Heterogeneous Duplications
Source: PLoS Biol. 2016 Dec 5;14(12):e2000618. doi: 10.1371/journal.pbio.2000618 (PMC5137868; doi:10.1371/journal.pbio.2000618)
Supplement: S3 Table — (PDF) [file pbio.2000618.s009.pdf]

S3 Table: Nature and number of *ace-1* copies in different mosquito genotypes.

Mosquito	Genotypes	Number of *ace-1* copies	
		S	R
**Strains**			
KisumuP	SS	2	0
Acerkis	R^3R^3	0	6
Acerduplikis	DD	2	2
AgRR5	R^5R^5	0	10
**F1 offspring from cross between strains**			
Acerkis x KisumuP	R^3S	1	3
Acerduplikis x Acerkis	DR^3	1	4
Acerduplikis x KisumuP	DS	2	1
